# The Impact of Corneal Oedema on UV Light Transmission: An Experimental Study in Porcine Eyes

**DOI:** 10.3390/jcm13237228

**Published:** 2024-11-28

**Authors:** Celeste Briceno-Lopez, Mª Carmen García-Domene, Cristina Peris-Martínez, Mª Jose Luque-Cobija, Mª Amparo Díez-Ajenjo, Neus Burguera-Giménez

**Affiliations:** 1 Department of Optics and Optometry and Vision Sciences, Faculty of Physics, Universitat de València, Dr. Moliner, 50, Burjassot, E-46100 Valencia, Spain; m.carmen.garcia-domene@uv.es (M.C.G.-D.); maria.j.luque@uv.es (M.J.L.-C.); amparo.diez@uv.es (M.A.D.-A.); neus.burguera@uv.es (N.B.-G.); 2 Cátedra Alcon—FOM—UVEG, Universitat de València, Dr. Moliner, 50, E-46100 Burjassot, Spain; 3 Anterior Segment and Cornea and External Eye Diseases Unit, Fundación de Oftalmología Médica, Av. Pío Baroja, 12, E-46015 Valencia, Spain; cristina.peris@fom.es; 4 Surgery Department, Faculty of Medicine, Universitat de València, Av. Blasco Ibáñez, 15, E-46010 Valencia, Spain

**Keywords:** corneal oedema, UV light transmittance, porcine eyes, corneal thickness, endothelial cell density

## Abstract

**Background/Objectives:** Corneal oedema is known for changing the cornea’s optical properties, particularly its ability to transmit ultraviolet (UV) light, which is crucial for visual clarity and eye health. This study explores how changes in corneal thickness in oedematous states affect UV light transmission. **Methods:** This study included 107 porcine eyes with artificially induced corneal oedema. Corneal thickness (CCT) was measured histologically, UV transmittance was assessed using a UV/VIS spectrometer, and endothelial cell parameters were evaluated with specular microscopy. Statistical analyses included the Kruskal–Wallis test, Mann–Whitney U test, and Spearman’s correlation. **Results:** The findings indicated a significant increase in CCT in oedematous corneas at 24 and 48 h post extraction compared to controls, with median CCT values of 816.59 ± 139.71 μm for controls, 1022.40 ± 234.48 μm at 24 h, and 1074.21 ± 220.83 μm at 48 h (*p* < 0.001). UV transmittance (395–280 nm) decreased substantially, dropping from 50.79 ± 7.65% in controls to 43.24 ± 5.35% at 24 h and 39.66 ± 6.51% at 48 h (*p* < 0.001). There was a significant negative correlation between CCT and UV transmittance (ρ = −0.346, *p* < 0.001). Endothelial parameters showed notable changes: maximum cell area (Area_MAX_) decreased at 24 and 48 h, while endothelial cell density (ECD) increased at 24 h. **Conclusions:** Our study found a substantial inverse link between CCT and UV light transmission in oedematous corneas, highlighting the importance of UV protection, especially in individuals who are prone to recurrent oedema. Changes in CCT and endothelial measures, such as Area_MAX_ and ECD, are useful signs of corneal integrity. However, the study’s small sample size and potential tissue modifications during processing need more research with bigger, in vivo samples to corroborate these findings and improve therapeutic use.

## 1. Introduction

Accurate corneal thickness assessment forms one of the cornerstones in ophthalmology, providing critical insights for a range of clinical applications from monitoring glaucoma progression to refractive surgical planning. Techniques such as Anterior Segment Optical Coherence Tomography (AS-OCT), specular microscopy, and ultrasound pachymetry (USP) have been demonstrated to be valid for the measurement of central corneal thickness (CCT) in normal corneas [1,2,3]. However, in thicker, oedematous corneas, many of these techniques are rendered less useful due to inherent methodological limitations, such as the use of algorithms and mathematical models which assume a constant value for the refractive index to estimate the parameters being measured [2,4,5,6,7,8,9].

The assessment of oedematous corneas poses several challenges. Not only does the marked change in corneal thickness resulting from fluid accumulation affect optical properties like transmittance, both of which directly influence the quality of vision and, by extension, the quality of life of a patient [10,11], but such hydration of the cornea itself is one of the most important yet most commonly overlooked elements. As the cornea becomes oedematous, the light beams passing through the cornea immediately have both their normal path and refractive index altered [12]. This assumes a constant index of refraction from fixed devices making the measurements, which is now compromised. It is important to recognise this site for implications providing proper measurement when assessing the magnitude of diseases like glaucoma, keratoconus, or corneal oedema [13,14,15].

Porcine eyes are a very good model system because of their similarities in structure and physiology to human eyes, in particular with respect to weight, size, and ocular fluid volumes. The scleral thickness and biomechanical properties of the porcine eye also match well with the human counterpart, thus further extending into modelling of surgical procedures and pharmacokinetic profiles [16]. It is, however, important to remember that permeability across the porcine cornea is low due to a thicker epithelium [17,18,19]. On the other hand, direct microscopic measurements are more accurate since they do not depend on the refractive index variable in oedematous corneas, unlike many optical methods. Similarly, ultrasound pachymetry does not rely on the refractive index but can be significantly affected by media opacities, such as those occurring in advanced stages of corneal oedema, which may compromise measurement reliability. This gives an uninterrupted view of the structure of the cornea with no incidence of the error introduced by indirect pachymetry’s assumed standard corneal refractive index of 1.3371.

The interplay of light transmittance and corneal oedema is a very critical and less explored domain within ophthalmology. Understanding how corneal oedema affects UV transmission is especially essential because the cornea protects deeper ocular structures from UV damage. Oedema-induced structural alterations, such as disorganised collagen fibrils and increased moisture, have been demonstrated in studies to drastically impair the cornea’s ability to filter hazardous wavelengths. These modifications could aggravate oxidative stress and cellular damage, notably in the endothelium [20,21]. In particular, there is a lacuna in the literature as far as directly determining the light transmittance properties related to corneal thickness in oedematous states, and this is where the study comes into play. Further, these studies could be applied in diseased conditions, such as uveitic inflammatory injury, where changes in translucency exacerbate the pathological condition [11,22]. Therefore, advanced imaging and precise light measurement techniques are up front, along with sophisticated models that mirror the physiological conditions of the human cornea with accuracy.

The presented investigation determines the impact of corneal oedema on light transmission by correlating histologically measured CCT with ultraviolet light transmittance and endothelial cell parameters in ex vivo porcine eyes. The relationship between increasing corneal thickness, altered endothelial morphology, and reduced optical clarity is studied in the present work to identify the most influential factor related to oedematous conditions leading to visual disablement. These results will be helpful in the improvement of the diagnostic precision and clinical management of corneal oedema, ultimately leading to better patient outcomes and preservation of vision.

## 2. Materials and Methods

In this experimental study, we used animal corneas that had been subjected to controlled oedematous processes. In total, 107 porcine eyes were included in this work. This work utilised porcine eyes opportunistically collected from animals that had been culled for agricultural purposes unrelated to this work and were not deliberately euthanized for this work. Eyes were enucleated at the abattoir by trained operators and then stored in containers kept at 4 °C in order to preserve corneal structural and functional integrity, as this temperature reduces metabolic activity and enzymatic degradation, delaying autolysis and keeping tissue hydrated. This approach, commonly employed in ophthalmic research, provides accurate measurements of parameters including UV transmittance and corneal thickness, in line with previous studies [21,23]. The eyes arrived on a Tuesday at about 11.00 a.m., approximately 5 ± 1 h after the pigs had been sacrificed. For immediate analysis, samples from four randomly selected eyes were analysed. Experimental groups’ eyes were kept at 24 and 48 h post extraction in a refrigerator at 4 °C in a humid chamber to preserve and evaluate the swelling process [24,25,26,27,28]. Random selection of the eyes was repeated for the eyeballs in the experimental groups. All the measurements were performed under the acceptance of the local ethical committee approval at Fundación de Oftalmología Médica (Approval Code: PI 119, May 2023). Inclusion criteria consisted of healthy corneas devoid of any pathological findings. Slit-lamp biomicroscopy was performed on the study eye before any testing in all groups to confirm the absence of pathology. Eyes were excluded if they exhibited any corneal irregularity that could influence measurement accuracy, such as epithelial damage from previous enucleation.

First, the corneal buttons were prepared within the laboratory setting. The samples were managed with one standard surgical set of instruments to minimise any differential variation in sample preparation. Using a trephine punch of 7.5 mm in diameter, the central corneal button was excised to include all the corneal layers for our analysis. The diameter was selected with regard to the necessity of measuring the central optical zone without possible artefacts arising from the inclusion of an edge of the sample in measurement. Transmittance measurements were made using a Perkin-Elmer Lambda 35 UV/VIS Spectrophotometer (PerkinElmer Life and Analytical Sciences, Shelton, CT, USA), spanning a spectrum from 280 to 1100 nm, which allows for an accurate spectral transmission originally within the UVB and IRA ranges of visible light. The precision of the device was up to 5 nm for wavelength and up to 2% for transmittance. Each corneal button was, in turn, sandwiched between two sapphire sheets, 7.5 mm in diameter and 0.4 mm thick, in a ‘sandwich structure’ as used in previous studies [23,29]. This assembly was then placed on a specially designed calliper, which was placed inside the integrating sphere of the spectrophotometer for the measurement of its transmittance.

An auto-zero calibration was performed before each session of measurement to ensure baseline accuracy. The calibration utilised the aluminium bracket and sapphire crystal. In the case of the transmittance measurements, the sapphire sheets in air were used as a blank reference.

It should be noted that reflection was not corrected on the first surface of the corneal tissues; therefore, the internal transmittance was not used but, instead, the measured transmittance was used. The integrating sphere used in the spectrophotometer collected all the light transmitted by the sample, referring to total transmission and not only the direct component. The average time to perform a single measurement of transmittance was around 2 min. Immediately following the transmittance measurements, each corneal button was individually placed in a sample container with Optisol-GS (Bausch & Lomb Incorporated, Bridgewater, NJ, USA) to maintain tissue viability and structural integrity. The specimens were then prepared for analysis using a Konan KeratoAnalyzer Specular Microscope (Konan Medical, Irvine, CA, USA), which offers magnified observation of the cell layer of the endothelium. The analysis was enabled using Debut Video Capture Software version 1.49 (NCH Software©, Greenwood Village, CO, USA), which captured nine high-resolution photographs of each cornea. In order to avoid similar sampling, the observed area within the endothelial layer in each image was changed to provide a better estimation of the endothelial cell density.

The endothelial variables measured included the following: area of cells averaged (AVE), maximum and minimum cell areas (Area_MAX_ and Area_min_), endothelial cell density (ECD), coefficient of variation (CV), and hexagonality (Hex).

During the whole experimental process, physiological saline solution was used in order to simulate the normal tear film and keep tissues hydrated. More precisely, we used B. Braun 0.9% sodium chloride solution (B. Braun SE, Melsungen, Germany) for parenteral use only in sterile vials of 10 mL each to avoid contamination and maintain isotonic conditions.

Histological analysis was started with the measurement of CCT. The fixed corneal buttons were processed by being transferred to 4% neutral buffered formalin, followed by a graded series of alcohols, and then embedded in paraffin wax. Uniformly sized thin sections, measuring 4 µm thick, were carefully sectioned from each paraffin-embedded sample. These were then subjected to haematoxylin and eosin staining to facilitate detailed histological analysis.

Some previous studies noted that formalin can cause tissue dehydration; hence, we adjusted each layer thickness by +5% [21,23]. It is important to point out that CCT measurements performed on fixed corneas may not accurately depict in vivo corneal thickness due to the effects of the lack of moisture and contraction induced during the fixation procedure, even though we corrected it. Previous research has shown that the use of formalin and paraffin can change the dimensions of corneal tissue, underestimating thickness when compared to fresh corneas or in vivo settings [21]. This factor represents a limitation in the direct application of fixed corneal outcomes to the clinical setting.

These sections were observed under a Leica microscope CTR5000 (Leica Microsystems GmbH., Wetzlar, Germany) to evaluate any abnormality in the integrity or tissue surrounding the measurement site in the cornea. The scaled photographs of sections under a light microscope were taken using a high-resolution camera by an experienced laboratory technician. Consequently, observation was made at 10×/20× magnification, whichever was suitable for the size of the sample. A total of nine different sets of measurements were made on each cornea to ensure the most accurate quantification of CCT. The image processing was very strict, with an error exertion for measurement of the thickness of the tissue as low as 0.01 µm. The transportation and storage of the corneal buttons were in a dry, light-protected area, hence guaranteeing the maintenance of their integrity till they were sectioned.

In fact, such thorough preparation and systematic microscopic examination were necessary for ensuring that our histological findings were reliable.

The results of each instrument were compared for measurement reproducibility using the Spearman correlation coefficient; thus, measurements of each session were averaged for each of the study groups.

### Statistical Analysis

All datasets underwent normality testing using the Shapiro–Wilk test, as neither of the groups had a sample size exceeding 50. Consequently, nonparametric statistical comparisons were conducted. SPSS for MacOS Version 28.0.1.1 (SPSS Inc., Chicago, IL, USA) was utilised for statistical analysis. The *p*-values in this article were adjusted to account for possible type I errors due to multiple comparisons by using the Bonferroni adjustment factor. The level of significance considered in all analyses was α < 0.05.

The observed histology measured the CCT directly. In addition, the corneal transmittance and endothelial parameters were measured. The study population was divided into three groups: ‘fresh’ eyes, serving as the control group, with non-oedematous corneas; eyes whose measurements were taken 24 ± 2 h after extraction; and eyes measured 48 ± 2 h post extraction. The main objectives were to observe the process of swelling of the cornea and analyse changes in the thickness of the cornea, the morphology of endothelial cells, and light refraction through transmittance. The entire study was conducted by one observer, and the data did not meet the criterion of a parametric distribution.

A Kruskal–Wallis test was conducted to analyze the differences in pachymetry, transmittance, and endothelial parameters among fresh eyes and eyes stored for 24 and 48 h. This test was compared to identify relevant differences inside the values of CCT regarding light refraction—that is to say, transmittance—and physiological modification of the endothelium. Where differences were significant in the previous step, post hoc analyses using nonparametric tests—either the Mann–Whitney U test or Wilcoxon test—were conducted to specify which particular group comparisons exhibited significant differences. Pairwise comparisons were conducted through application of the Mann–Whitney U test in the post hoc test. The Bonferroni correction was applied because conducting multiple tests increases the risk of type I error.

The adjustment performed here imposes an α level of 0.0167, which corresponds to a 99.98% Confidence Interval. That is, it ensures that the probability of rejecting a true null hypothesis among all the tests does not exceed the conventional alpha level of 0.05.

The correlation between the variables of CCT, transmittance, and endothelial cell parameters was studied using Spearman’s correlation coefficient. First, we studied the correlation of these variables amongst themselves. Later on, their association with the study groups was analysed. This analysis was carried out in order to explore, from these variables, the presence of a relationship among them and their respective associations with their study groups. The important thing is the sign and magnitude of this coefficient in the estimate of agreements, whose result varies between 0 and 1, where 1 is the maximum agreement between the devices.

## 3. Results

### 3.1. Descriptive Statistics

A total number of one hundred and four eyes were assessed in this study. As control groups for this study, we utilised the enucleated eyes from the same day (n = 34). All the cases, in fact, had oedema both at 24 and at 48 h after enucleation. Descriptive statistics of the variables include central tendencies and dispersion measures as described in Table 1. The normality tests showed that our sample sets were nonparametric.

### 3.2. Visual Data Exploration

Scatter plots for selected pairs of variables follow in the below graphical formats. These graphs pertain only to those combinations of variables which demonstrated statistically significant relationships from our inferential analysis. This selective approach allows the information to visually align with our statistically significant results and thus provide a stronger, concise presentation of key findings.

#### Histology

**Figure 1:**
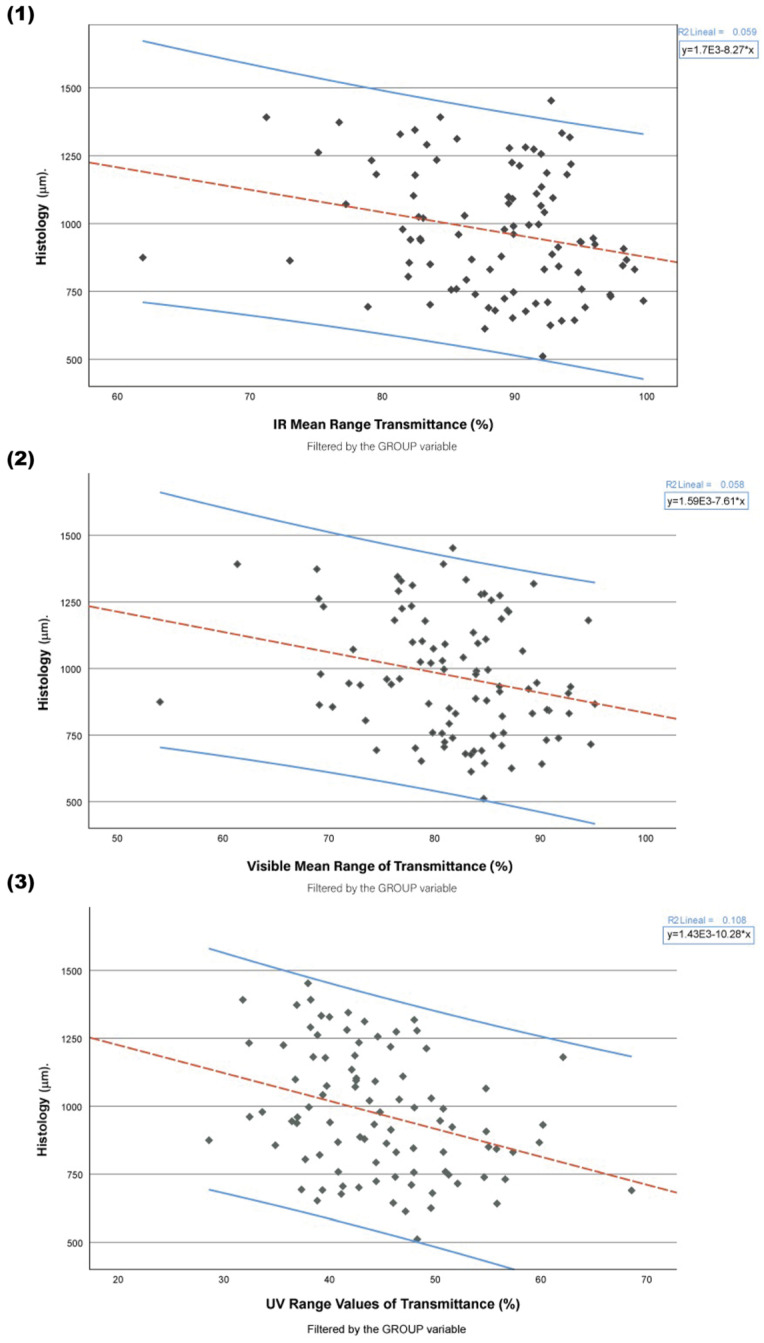
Scatter plots illustrating the correlation between histological measurements of central corneal thickness (CCT) and average light transmittance across different energy spectra. (**1**) Correlation within the infrared (IR) spectrum (1100–700 nm); (**2**) correlation within the visible (Vis) spectrum (695–400 nm); (**3**) correlation within the ultraviolet (UV) spectrum (395–280 nm). The regression line (blue) indicates the trend for each energy range, with the shaded area representing the 95% Confidence Intervals (CIs). Linear regression equations and coefficient of determination (R^2^) values are displayed in the top right corner of each plot. The ‘Group’ variable (control, 24 h oedema, and 48 h oedema) serves as the filter for data stratification.

Scatter plots were developed to illustrate the association between histology measures of CCT and light transmittance across various energy spectra. We isolated three distinct spectrums of light energy, infrared, visible light, and ultraviolet, as shown in Figure 1. This gave us a much clearer visual and allowed us to understand the workings in each spectral portion.

**Figure 2:**
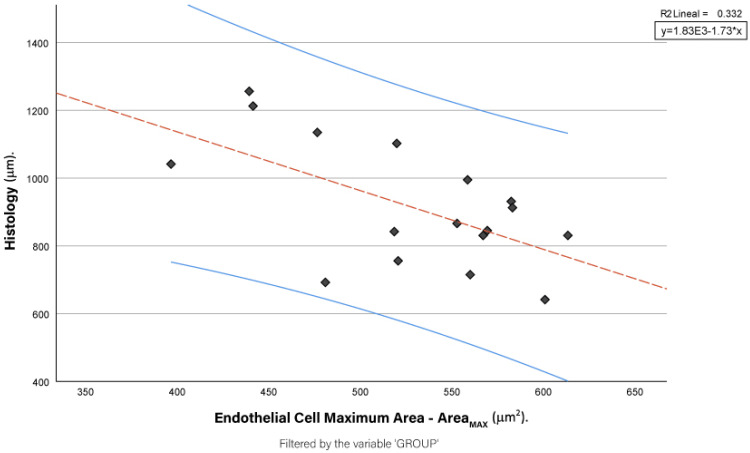
Scatter plot showing the correlation between histological measurements of central corneal thickness (CCT) and maximum endothelial cell area (Area_MAX_) across all study groups (control, 24 h oedema, and 48 h oedema). The regression line (blue) indicates the trend, with the shaded area representing the 95% Confidence Interval (CI). The linear regression equation and the R^2^ value are provided in the top right corner. The ‘Group’ variable is used to categorise the data by oedema duration.

Further, we analysed the correlation between histological measures of CCT and endothelial parameters. Among those, only the scatter plot between CCT and Area_MAX_ is shown here for its subsequent statistically significant Spearman’s Rho correlation (Figure 2).

**Figure 3:**
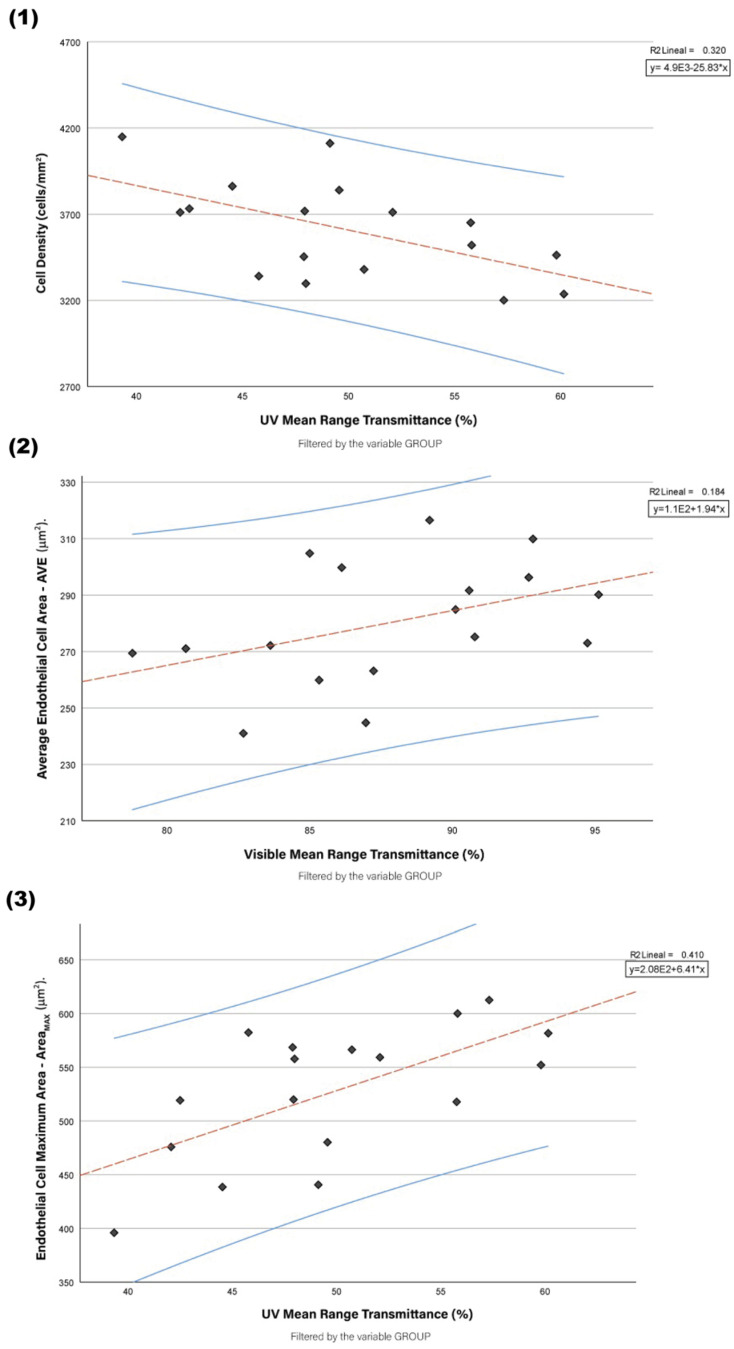
Scatter plots depicting the relationships between endothelial parameters and light transmittance across study groups. (**1**) Correlation between endothelial cell density (ECD) and UV transmittance mean; (**2**) correlation between average endothelial cell area (AVE) and visible light transmittance (Vis) mean (695–400 nm); (**3**) correlation between maximum endothelial cell area (Area_MAX_) and ultraviolet (UV) transmittance mean (395–280 nm). The regression lines (blue) show the trend for each correlation, with shaded areas representing 95% CI. The linear regression equation and R^2^ values are displayed for each plot. The ‘Group’ variable serves as the filter to distinguish between the control, 24 h oedema, and 48 h oedema groups.

In this case, by focusing on the energy spectrum, scatter plots were constructed to visually dissect in detail the relationship existing between average values of transmittance for each energy range and endothelial parameters. Figure 3 allows for a nuanced understanding of how variations in transmittance values correspond to changes in endothelial characteristics.

### 3.3. Inferential Analysis

#### 3.3.1. Correlation Analysis Between Variables

##### Transmittance—CCT

Spearman’s correlation coefficient was used to evaluate the relationship between the CCT measurements obtained through histology and the corresponding transmittance values for each study group.

In order to simplify the analysis, we aggregated data into the average for categories of transmittance, using a standard classification of light into infrared, visible, and ultraviolet regions between 1100 and 700, between 695 and 400, and between 395 and 280 nm, respectively. Because of this, we used average measurements within these specified ranges to calculate Spearman’s correlation coefficient, Rho (Table 2).

##### Transmittance—Endothelial Parameters

To further quantify the changes in corneal optics along with the morphology of the endothelium during the procession of oedema, Spearman’s rank correlation coefficient was calculated between the values of transmittance and those of endothelial cell metrics such as ECD, CV, and Hex (Table 3). We would expect that, since the transparency of the cornea decreases with loss of function of the endothelial pump in oedema, there is a monotonic relation between optical transmittance and the indicators of the status of endothelial health.

Specifically, such reduced transmittance would correlate with indices of endothelial damage such as a lower ECD and Hex and a higher CV. Spearman’s nonparametric correlation was adopted because the nature of the transmittance is measured on a continuous linear scale, while such metrics as CV are non-normally distributed. The analysis of these kinds of correlations allows insight into how the interacting optical and endothelial properties change over time as oedema develops.

##### CCT—Endothelial Parameters

The relation between CCT and ECD was also analysed to further characterise the structural changes occurring in the course of corneal oedema. CCT has traditionally been a strong predictor of the state of corneal oedema and fluid accumulation within the stroma. The pathogenesis of oedema is powered by the endothelial pump, and it is for this reason that ECD is expected to be decreasing.

However, the observed increase in ECD for the oedematous groups represents a trend opposite to that normally expected. This may thus point to deficiencies inherent in these indirect methods of measurement. The discrepancy thus calls for caution when interpreting ECD values obtained under such conditions. In accordance, we expect that as oedema progresses, these two variables will relate inversely to each other—for progressively higher CCT values, lower ECD values will be obtained. The CCT was further correlated with the endothelial morphometry measures, including, but not limited to, CV and Hex, in order to associate the stromal thickness with both polymegathism and pleomorphism.

Among the endothelial parameters, only Area_MAX_ showed a significant inverse correlation with CCT, with a Spearman coefficient of ρ = −0.525 (*p* = 0.031). This suggests that increased CCT is associated with a decrease in Area_MAX_, highlighting potential structural changes in corneal endothelial cells as oedema progresses.

### 3.4. Group Comparisons

#### 3.4.1. Central Corneal Thickness

The comparison of histology between the study groups showed a statistical difference in the mean CCT for control versus 24 h and between control and 48 h at *p* = 0.003, H = −24.161 and *p* = 0.000, H = −31.324, respectively. Only 24 h versus 48 h did not show any significance (see Table 4).

In post hoc analysis, the Mann–Whitney U test demonstrated statistically significant differences among groups. The value of CCT as obtained from the histological methods did not differ significantly between the 24 h and 48 h groups.

#### 3.4.2. Transmittance

**Figure 4:**
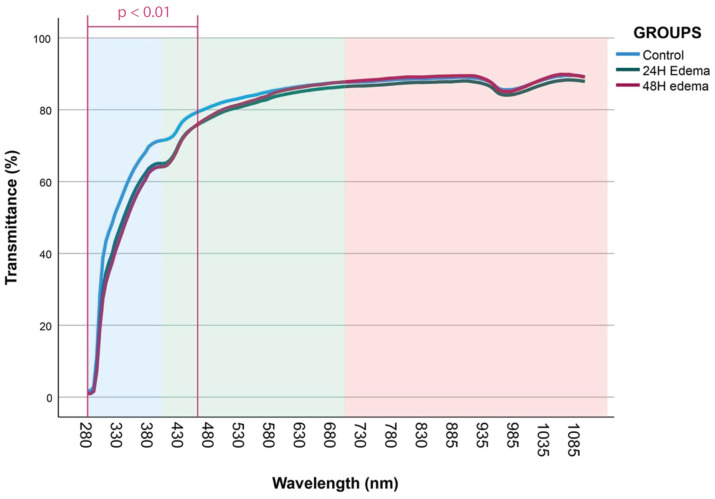
Graphical representation of the percentage transmittance measured across the control and study groups (control, 24 h oedema, and 48 h oedema) for three spectral regions: infrared (IR) wavelengths (1100–700 nm, highlighted in red), visible light (Vis) wavelengths (695–400 nm, in green), and ultraviolet (UV) wavelengths (395–280 nm, in blue). Statistical significance was assessed using the Kruskal–Wallis test, with subsequent Mann–Whitney U post hoc tests for pairwise comparisons between groups. The *p*-values were adjusted with the Bonferroni correction, setting a threshold of *p* < 0.0167 for multiple comparisons.

Transmittance showed significant difference in short wavelengths across the range from 460 nm to 295 nm (Table 5). However, a comparison of averages of the transmittance started showing significance at 450 nm to 295 nm, and this always happened for the same two out of three comparisons (control vs. 24 h and control vs. 48 h; see Figure 4). All 24 h vs. 48 h analyses did not show any statistical difference.

#### 3.4.3. Endothelial Parameters

Only medians of endothelial parameters for each of the groups were compared. The endothelial variables measured included the mean cell area AVE, Area_MAX_ and Area_min_, ECD, CV, and Hex. Distribution comparison was statistically significant for AVE (*p* = 0.019), Area_MAX_ (*p* = 0.010), and ECD (*p* = 0.019) between the control vs. 24 h groups.

On the other hand, the pairwise comparison post hoc test revealed statistical significance only in the control group vs. the 24 h group for AVE, Area_MAX_, and ECD, with *p*-values of *p* = 0.014, z = −2.373; *p* = 0.008, z = −2.556; and *p* = 0.017, z = −2.373, respectively. This analysis, however, also revealed a significant difference for the Area_MAX_ when the 48 h group was compared to the control group at *p* = 0.045, z = −2.030 (shown in Table 6).

### 3.5. Multiple Regression Analysis

A stepwise multiple regression was also carried out to assess the overall effect of several independent variables on UV light transmission through the cornea. The first model included histology and all the endothelial parameters as independent variables. The stepwise regression model identified Area_MAX_ as the only significant predictor of UV transmittance. This model explained a correlation coefficient, R = 0.640, showing a moderate association between Area_MAX_ and the dependent variable, UV transmittance, AVE_UV_.

The R^2^ determined was 0.410, which shows that 41.0% of the variability in UV transmittance can be accounted for by Area_MAX_. The Adjusted R-squared was 0.371, which is a small reduction when accounting for the total number of predictors in this model. This would be indicative of the model remaining reasonably robust when Area_MAX_ is the sole predictor. The standard error of the estimate was 4.933; this gave an indication of the actual attained accuracy of the model.

Also, ANOVA of the regression model showed that the sum of squares of regression was 253.899 with 1 degree of freedom and a mean square of 253.899. The F-value was 10.433, with a significance level of 0.006, showing that the model is statistically significant (*p* < 0.05). The constant had a value of 16.117, t = 1.532, with a significance level of *p* = 0.146; hence, it is not statistically significant. For Area_MAX_, the coefficient was 0.064, t = 3.230, and *p* = 0.006; hence, Area_MAX_ is a significant predictor of UV transmittance at *p* < 0.05. The standardised coefficient (Beta) for Area_MAX_ was 0.640; hence, it has a moderate positive relation to UV transmittance.

The histology, AVE, Area_min_, CV, ECD, and Hex excluded variables in this case that did not significantly contribute to the model and thus were excluded during the stepwise regression process. This is a method of stepwise regression that is an iterative addition or removal of predictors from the model based on their statistical significance for explaining variance in the dependent variable.

No problems of collinearity were shown from the collinearity diagnostics, as highlighted by the fact that Tolerance was equal to 1.000 and the variance inflation factor (VIF) for Area_MAX_ equalled 1.000, as shown in Table 7, ascertaining that the predictor included in the model does not hold redundancy with other predictors.

## 4. Discussion

Our study examined the effects on empirically induced corneal oedema in porcine eyes upon corneal thickness (CCT), UV light transmission, and endothelial cell morphology. CCT, an essential metric in ophthalmology [2,24,25,26], is crucial for reliable intraocular pressure (IOP) measurements [15,27] and refractive surgery planning [28]. The use of a porcine model, combined with direct histological CCT evaluation, overcomes the limitations inherent in clinical approaches for evaluating oedematous corneas [5,24]. The significant CCT increase within 24 h post extraction highlights the cornea’s physiological limits in fluid absorption capacity under oedematous conditions [29,30,31,32].

Our results showed an inverse relationship between CCT and the transmission of light, particularly in the UV region. This inverse relationship between the two parameters suggests that the greater the thickness of the cornea, the less will be the amount of energy transmitted through the cornea, thereby affecting conditions like cataracts and macular degeneration [33,34]. These results suggest that the clinical impact of corneal oedema is associated with UV protection and reaches a critical plateau at 48 h, which could serve as an indicator of severe corneal swelling. However, it is important to consider that fixation-induced tissue contraction could have influenced the absolute values of CCT, as suggested by Peris-Martínez et al. [23] and Kolozsvári et al. [21]. While we applied a +5% correction factor to mitigate this effect, the possibility of residual discrepancies remains a limitation.

In contrast, reduced UV transmission in oedematous corneas suggests a transient protective effect, as greater corneal thickness reduces UV exposure to deeper layers. However, while this restriction appears to be beneficial, it fails to address long-term weaknesses. Oedema-induced swelling changes the cornea’s structural organisation, particularly in the stroma, where increased spacing between collagen fibrils improves light dispersion and decreases UV absorption, exposing deeper layers to damaging wavelengths [35,36]. Although Bowman’s layer, the most effective UV absorber, is important, it frequently cannot compensate for structural deficiencies, increasing the risk of UV-induced oxidative damage to the endothelium [20,21].

This risk is heightened by cumulative oxidative damage from UV exposure, which can cause keratitis and other epithelial diseases, especially when natural protective barriers are compromised [30]. Oedema-induced structural disorganisation, such as ‘lakes’ of fluid in the stroma, decreases optical quality by increasing light scattering and predisposes the cornea to permanent damage, including leukomas and scarring [35].

Furthermore, age-related decreases in UV filtering efficiency exacerbate these hazards [37,38]. Unlike the lens, which acquires a protective ‘yellow filter’ effect with age, oedematous corneas are unable to selectively filter damaging wavelengths [39]. Individuals with recurrent or chronic oedema must take preventive measures, such as UV protection, to avoid complications. These precautions are especially important for patients who are prone to radiation-induced keratopathies, as poor UV filtering can exacerbate the pathological condition.

The relationship of CCT with endothelial parameters, especially the negative correlation with Area_MAX_ [28,40,41,42], gives an idea about the structural changes in corneal oedema [28,43]. In contrast to other endothelium metrics, Area_MAX_ was found to be a significant predictor of UV transmittance in the regression analysis, accounting for 41.0% of the variation. This demonstrates its sensitivity to morphological changes in the endothelium layer during oedematous conditions, where cell expansion is a compensatory mechanism for maintaining functionality despite stress. These structural alterations increase light scattering because bigger cell areas disrupt the organised lattice-like structure of collagen fibrils and other stromal components [43]. Such disruptions not only lower corneal transparency, but also reduce the cornea’s UV radiation protection by changing how light is dispersed and absorbed [35,41]. Area_MAX_ shows more direct and specific cellular responses to physiological stress than metrics such as endothelial cell density (ECD) or hexagonality, which may explain its significance in our research [40,41]. However, because the results are based on ex vivo models, additional research is needed to validate these findings and investigate their clinical relevance in controlling corneal transparency and structural integrity during oedema.

A reverse relation between CCT and ECD, as an indicator of the function of the endothelial pump, was not fully observed [40], probably because of the limited sample size or as a consequence of tissue alterations during processing [21,23]. The lack of CCT correlations with AVE, ECD, CV, and Hex indicates that such a relationship with the endothelium exists in so intricate a manner that further research with more measurements is warranted to reach a complete understanding of the relationship existing between corneal thickness and endothelial health.

The transmittance records showed significant reductions in the average transmittance percentage of the oedematous corneas compared with controls, particularly in the UV spectral region; this suggested that such corneal swelling could limit visual quality through alterations in the optical properties [10]. Our analysis showed that, for the shorter wavelengths, there were significant differences in the transmittance percentage between controls and post-extraction groups, as depicted in Table 3. Transmittance for all wavelengths was reduced by 7.55% and 11.13%, respectively, in the 24 h and 48 h groups when compared to the control group (both *p* < 0.001). There was also a 3.19% reduction versus controls for 24 h in the visible range (*p* = 0.031). As such, the penetration of shorter wavelengths does raise concerns of ultraviolet light reaching the posterior pole [44,45]. Energy transmission through the cornea reduces with the increase in CCT, and such reduction may have some consequences regarding diseases like cataracts and macular degeneration [11,22,29,45].

Simple linear regression analyses were conducted to quantify the relationship between histological measures of CCT and absorption coefficients for each spectral wavelength band: IR, visible, and UV. In fact, it makes it safe for the significant predictive models for UV and visible light transmittance through corneal tissue to assume that possible changes in CCT may be responsible for changes in the absorption coefficient across these different ranges—a fact that is quite critical in furthering the light—tissue interaction in conditions of oedema [23,46,47,48].

Significant endothelial variations were recorded in AVE, ECD, CV, and Hex. The Spearman correlation shows an inverse CCT–transmittance relationship, especially within the UV range, suggesting that increased thickness in the cornea acts as a barrier to the passage of light [11].

We significantly enriched our analysis by applying a multiple regression model, custom-designed to probe into the interaction of several corneal factors acting in concert on the variation in optical properties under oedema conditions. The comprehensive approach has also confirmed that CCT is in reverse proportion with light transmission and has shown the great predictive power of several corneal parameters on shifting the absorption coefficients in the UV and visible spectra.

By including several variables in the analysis, we were able to recognise latent complexity: how corneal thickness coupled with structural changes at the level of layers and the endothelial health of the cornea cumulatively acts on its optical properties. Indeed, this points to the necessity of clinical management strategies that address not just one parameter but the multifactorial interaction defining the pathologic state of the cornea.

Our study further explores changes in the thickness of each layer of the cornea in oedema. Large variations existed between Bowman’s layer, stroma, and endothelium thickness between the groups. This may suggest that such changes are responsible for the above-described changes in the optical and transmittance properties of the oedematous cornea [46,49,50]. A detailed understanding of mechanisms that lead to the deterioration of visual quality may emerge from comprehensive analysis of such layers.

Our data show a strong relationship between increased CCT in oedematous corneas and lower UV transmission, implying that while this reduction provides temporary protection, it highlights the importance of UV protection once the oedema has resolved. Individuals with temporary corneal thickening may be more vulnerable to UV-induced damage, underlining the need for precautions.

This study underlines the clinical significance of corneal oedema, emphasising the importance of UV protection and the 48 h plateau as indicators of severe corneal oedema, which can result in endothelial cell death, reduced transparency, and ultimately endothelial decompensation [51,52]. Early management is critical because the ion pump mechanism cannot maintain stromal deturgescence at maximum fluid retention, leading to permanent changes such as “oil droplet” keratopathy and changed corneal curvature [53,54].

The study also emphasises the necessity of including several corneal characteristics into regression-driven models to improve clinical prediction and management skills. However, constraints such as a small sample size and potential tissue alterations during processing show the importance of conducting additional studies with bigger, in vivo samples to confirm these findings and investigate their clinical implications. Finally, maintaining corneal transparency and functionality necessitates a multifaceted approach that considers both structural changes and UV-related hazards.

## Figures and Tables

**Table 1:** Descriptive statistics for central corneal thickness (CCT), light transmittance, and endothelial parameters measured across the study groups (control, 24 h oedema, and 48 h oedema).

	Control Group (n = 34)		24 h Oedema (n = 35)		48 h Oedema (n = 35)	
	Median	Std. Dev.	Median	Std. Dev.	Median	Std. Dev.
Histology						
CCT (μm)	816.59	139.71	1022.40	234.48	1074.21	220.83
Transmittance						
IR (1100–700 nm)	89.51	6.15	89.55	6.54	91.75	7.16
Visible (695–400 nm)	83.86	6.95	80.67	6.60	82.65	7.82
UV (395–280 nm)	50.79	7.65	43.24	5.35	39.66	6.51
Endothelial parameters						
AVE (μm^2^)	309.88	14.93	266.24	19.95	275.17	20.11
Area_MAX_ (μm^2^)	600.13	24.21	499.74	47.91	559.33	67.31
Area_min_ (μm^2^)	101.75	10.67	111.06	15.70	103.88	10.04
ECD (cells/mm^2^)	3237.00	160.58	3786.26	267.01	3650.83	276.95
CV (%)	35.33	1.50	35.76	2.72	35.33	3.25
Hex (%)	52.67	5.85	49.53	3.72	48.17	5.62

Data are presented as median ± standard deviation for each group. Transmittance values are shown for three spectral regions: infrared (IR: 1100–700 nm), visible light (Vis: 695–400 nm), and ultraviolet (UV: 395–280 nm). Endothelial parameters include mean cell area (AVE), maximum cell area (Area_MAX_), minimum cell area (Area_min_), endothelial cell density (ECD), coefficient of variation (CV), and hexagonality (Hex).

**Table 2:** Spearman correlation coefficients (ρ) between central corneal thickness (CCT) and light transmittance across infrared (IR), visible light (Vis), and ultraviolet (UV) spectral ranges.

		Spearman’s Rho	Sig. (2-Tailed)
Histology	IR_mean_	−0.243	**0.020**
	Visible_mean_	−0.257	**0.013**
	UV_mean_	−0.346	**<0.001**

All correlations were statistically significant (*p* < 0.05) and indicate an inverse relationship between CCT and transmittance. The coefficients were calculated using Fisher’s r-to-z transformation for accuracy, and the standard errors are adjusted according to the method by Caruso and Cliff for the dataset’s characteristics. Statistically significant results are marked as bold format.

**Table 3:** Spearman correlation analysis between transmittance values and endothelial parameters for each spectral range.

		Spearman’s Rho	Sig. (2-Tailed)
AVE (μm^2^)	IR_mean_	0.417	0.096
	Visible_mean_	0.471	0.057
	UV_mean_	0.554	**0.021**
Area_MAX_ (μm^2^)	IR_mean_	0.458	0.064
	Visible_mean_	0.461	0.063
	UV_mean_	0.564	**0.018**
Area_min_ (μm^2^)	IR_mean_	0.017	0.948
	Visible_mean_	0.208	0.422
	UV_mean_	0.076	0.772
ECD (cells/mm^2^)	IRmean	−0.390	0.122
	Visible_mean_	−0.441	0.076
	UV_mean_	−0.529	**0.029**
CV (%)	IR_mean_	0.037	0.889
	Visible_mean_	0.142	0.586
	UV_mean_	0.189	0.468
Hex (%)	IR_mean_	−0.206	0.428
	Visible_mean_	−0.311	0.224
	UV_mean_	−0.132	0.613

Statistically significant correlations are highlighted as bold format, including average cell area (AVE), maximum cell area (Area_MAX_), and endothelial cell density (ECD), within the ultraviolet (UV) range. Correlation coefficients were calculated with Fisher’s r-to-z transformation, and standard errors were determined using Caruso and Cliff’s method, suitable for this dataset.

**Table 4:** Results of the Kruskal–Wallis test for group comparisons on central corneal thickness (CCT).

		Kruskal–Wallis				Post Hoc		
		Test Stat.	Std. Error	Sig.	Adj. Sig.	Mann–Whitney U	Z	Sig.
Histology (μm)	Control—24 h	−24.161	7.243	<0.001	**0.003**	286.000	−3.271	**<0.001**
	Control—48 h	−31.324	7.290	<0.001	**0.000**	195.000	−4.342	**0.000**
	24 h—48 h	−7.163	6.955	0.303	0.909	-	-	-

Post hoc pairwise comparisons were conducted with the Mann–Whitney U test, where significant differences were identified. A Bonferroni correction was applied to adjust for multiple comparisons, setting the significance level at α = 0.0167. Statistically significant results are marked as bold format.

**Table 5:** Kruskal–Wallis test results for light transmittance across study groups in three spectral regions: infrared (IR), visible light (Vis), and ultraviolet (UV).

		Kruskal–Wallis				Post Hoc		
		Test Stat.	Std. Error	Sig.	Adj. Sig.	Mann–Whitney U	Z	Sig.
IR (1100–700 nm)	Control—24 h	9.263	7.077	0.191	0.572	-	-	-
	Control—48 h	0.746	7.163	0.917	1.000	-	-	-
	24 h—48 h	−8.517	6.479	0.189	0.566	-	-	-
Visible (695–400 nm)	Control—24 h	14.794	7.077	**0.037**	0.110	279.000	−2.176	**0.031**
	Control—48 h	9.981	7.163	0.164	0.491	-	-	-
	24 h—48 h	−4.813	6.479	0.458	1.000	-	-	-
UV (395–280 nm)	Control—24 h	24.667	7.077	<0.001	**0.001**	164.000	−3.950	**<0.001**
	Control—48 h	32.848	7.163	<0.001	**0.000**	144.000	−4.073	**0.000**
	24 h—48 h	8.182	6.479	0.207	0.620	-	-	-

Post hoc pairwise analyses were conducted with the Mann–Whitney U test where significant differences were identified, and Bonferroni corrections were applied to maintain a significance threshold of α = 0.0167. Statistically significant results are marked as bold format.

**Table 6:** Comparison of endothelial parameters among study groups using the Kruskal–Wallis test.

		Kruskal–Wallis				Post Hoc		
		Test Stat.	Std. Error	Sig.	Adj. Sig.	Mann–Whitney U	Z	Sig.
AVE	Control—24 h	8.567	3.233	0.008	**0.024**	2.000	−2.373	**0.017**
	Control—48 h	5.257	3.126	0.093	0.278	6.000	−1.868	0.069
	24 h—48 h	−3.310	2.970	0.265	0.795	12.000	−1.286	0.239
Area_MAX_	Control—24 h	8.967	3.233	0.006	**0.017**	1.000	−2.556	**0.008**
	Control—48 h	5.943	3.126	0.057	0.172	5.000	−2.030	0.045
	24 h—48 h	−3.024	2.970	0.309	0.926	13.000	−1.143	0.297
Area_min_	Control—24 h	−1.033	3.233	0.749	1.000	13.000	−0.365	0.793
	Control—48 h	−0.914	3.126	0.770	1.000	16.000	−0.244	0.883
	24 h—48 h	0.119	2.970	0.968	1.000	21.000	0.000	1.000
CD	Control—24 h	−8.567	3.233	0.008	**0.024**	2.000	−2.373	**0.017**
	Control—48 h	−5.257	3.126	0.093	0.278	6.000	−1.868	0.069
	24 h—48 h	3.310	2.970	0.265	0.795	12.000	−1.286	0.239
CV	Control—24 h	−0.300	3.231	0.926	1.000	15.000	0.000	1.000
	Control—48 h	0.771	3.124	0.805	1.000	16.500	−0.163	0.916
	24 h—48 h	1.071	2.969	0.718	1.000	18.000	−0.429	0.733
Hex	Control—24 h	1.767	3.233	0.585	1.000	11.000	−0.730	0.533
	Control—48 h	1.314	3.126	0.420	1.000	16.000	−0.244	0.883
	24 h—48 h	−0.452	2.970	0.879	1.000	21.000	0.000	1.000

Where the initial test indicated significant differences, post hoc analyses were performed with the Mann–Whitney U test. A Bonferroni correction was applied to adjust for multiple comparisons, with α = 0.0167. Statistically significant results are marked as bold format.

**Table 7:** Coefficient table for the regression model predicting UV light transmittance, showing the relationship between maximum cell area (Area_MAX_) and UV transmittance.

Model	Unstandardized Coefficients (B)	Std. Error	Standardized Coefficients (Beta)	t	Sig.	Collinearity (Tolerance)	VIF
Constant	16.117	10.517		1.532	0.146		
Area_MAX_	0.064	0.020	0.640	3.230	**0.006**	1.000	1.000

The unstandardized coefficient (B) indicates the change in UV transmittance per unit increase in Area_MAX_. Collinearity statistics (Tolerance and VIF) confirm no multicollinearity in the model, while the standardized coefficient (Beta) shows a moderate positive relationship between Area_MAX_ and UV transmittance. Statistically significant results are marked as bold format.

## Data Availability

Data supporting the study’s conclusions are accessible from the corresponding author upon reasonable request. Due to ethical constraints and the nature of the experimental data, access is restricted to researchers affiliated with academic institutions for non-commercial use. During this investigation, no publicly archived datasets were used or generated.
